# Translational Dysregulation in Cancer: Molecular Insights and Potential Clinical Applications in Biomarker Development

**DOI:** 10.3389/fonc.2017.00158

**Published:** 2017-07-26

**Authors:** Christos Vaklavas, Scott W. Blume, William E. Grizzle

**Affiliations:** ^1^Department of Medicine, Division of Hematology/Oncology, University of Alabama at Birmingham, Birmingham, AL, United States; ^2^Department of Anatomic Pathology, University of Alabama at Birmingham, Birmingham, AL, United States

**Keywords:** cancer, messenger RNA translation, dysregulation, biomarker, biological tumor marker, eIF4E, translational machinery, neoplastic transformation

## Abstract

Although transcript levels have been traditionally used as a surrogate measure of gene expression, it is increasingly recognized that the latter is extensively and dynamically modulated at the level of translation (messenger RNA to protein). Over the recent years, significant progress has been made in dissecting the complex posttranscriptional mechanisms that regulate gene expression. This advancement in knowledge came hand in hand with the progress made in the methodologies to study translation both at gene-specific as well as global genomic level. The majority of translational control is exerted at the level of initiation; nonetheless, protein synthesis can be modulated at the level of translation elongation, termination, and recycling. Sequence and structural elements and epitranscriptomic modifications of individual transcripts allow for dynamic gene-specific modulation of translation. Cancer cells usurp the regulatory mechanisms that govern translation to carry out translational programs that lead to the phenotypic hallmarks of cancer. Translation is a critical nexus in neoplastic transformation. Multiple oncogenes and signaling pathways that are activated, upregulated, or mutated in cancer converge on translation and their transformative impact “bottlenecks” at the level of translation. Moreover, this translational dysregulation allows cancer cells to adapt to a diverse array of stresses associated with a hostile microenviroment and antitumor therapies. All elements involved in the process of translation, from the transcriptional template, the components of the translational machinery, to the proteins that interact with the transcriptome, have been found to be qualitatively and/or quantitatively perturbed in cancer. This review discusses the regulatory mechanisms that govern translation in normal cells and how translation becomes dysregulated in cancer leading to the phenotypic hallmarks of malignancy. We also discuss how dysregulated mediators or components of translation can be utilized as biomarkers with potential diagnostic, prognostic, or predictive significance. Such biomarkers have the potential advantage of uniform applicability in the face of inherent tumor heterogeneity and deoxyribonucleic acid instability. As translation becomes increasingly recognized as a process gone awry in cancer and agents are developed to target it, the utility and significance of these potential biomarkers is expected to increase.

## Introduction

Although our understanding of transcriptional regulation and dysregulation in cancer has significantly advanced over the last decade, comparatively little is known about dysregulation of gene expression at the translational level. However, it is recognized that gene expression is extensively and dynamically modulated at the translational level accounting to a large extent for the discrepancies between messenger RNA (mRNA) and matching protein levels. Although transcript levels have been traditionally used as a proxy of the protein abundance in a cell, genomic-scale studies have shown that the latter is predominantly controlled at the posttranscriptional level and especially translation (1). Translation represents a more proximal level of control, allowing the cell to adapt swiftly to stress conditions by modulating protein synthesis from an existing pool of mRNAs, unlike the process of transcription which mediates more permanent changes in cell physiology or fate (2). Indeed, studies have shown that in response to stress, changes in translation precede and are of a greater magnitude than changes in transcription (3). In fact, immediate changes in the translation of transcripts encoding for transcription factors steer the later-appearing and more permanent changes in transcription (3). Translation and translational control is emerging as a critical nexus in mediating adaptive stress responses allowing cancer cells to overcome a diverse array of stress conditions imposed on them by the tumor microenvironment, immune recognition, their own continuous replication, and therapeutic modalities. Simultaneously, qualitative and quantitative changes in the translational machinery are critical mediators that carry out the transformative impact of oncogenes and oncogenic signaling.

This review provides an overview of translation and the regulatory mechanisms that govern it in normal cells. We discuss ways whereby translation becomes dysregulated in cancer and how oncogenic insults uniformly converge at the level of translation and modulate the translational landscape. Indeed all factors involved in carrying out the process of translation, from the mRNA which provides the template of the protein to be synthesized, the components of the translational machinery tRNA and ribosomes, to the multiple and diverse proteins that interact with the mRNA, can be perturbed in cancer. Coordinate changes in any or all of these elements may contribute significantly to the malignant behaviors of the transformed cells. In this context, mediators or components of translation that are aberrantly expressed or modified in cancer arise as biomarkers with potential prognostic or predictive significance (Table 1; Table S1 in Supplementary Material). Although none of these factors has reached broad clinical applications, extensive preliminary work has correlated aberrations in the components of translation with clinical outcomes. Since translation is a downstream process into which diverse arrays of oncogenic pathways converge, one potential advantage of these biomarkers is their fairly uniform perturbation, even in the face of tumor heterogeneity underpinned by genomic instability and multiple, redundant, interwoven, and bypassing signaling cascades. This advantage has been exemplified in BRAF(V600)-mutant melanoma, colon, and thyroid carcinoma, whereby the formation of eIF4F translation initiation complexes was the common point of convergence of multiple pathways that conferred resistance to targeted anti-BRAF, anti-MEK, and anti-BRAF plus anti-MEK drug combinations (4).

**Table 1:** Common malignancies in which the utility of factors or regulators of translation has been explored as potential biomarkers.

Organ of origin	Factor
Breast	hnRNP A1 (5), Y-box binding protein 1 (YB-1) (6), HuR (7, 8), IGF2BP3 (9), eIF4E (10–15), 4E-BP1 (16), eIF4AI (12), eIF4B (12), eIF4G (17), PCDC4 (12), fibrillarin (FBL) (18), elongator acetyltransferase complex subunit 3 (ELP3) and CTU1/2 (19)
Lung	YB-1 (20), HuR (21, 22), PCDC4 (23), tRNA isopentenyltransferase 1 (TRIT1) (24)
Gastrointestinal tract	hnRNP A1 (25), YB-1 (26–28), HuR (29–31), IGF2BP3 (32–35), eIF4E (36, 37), 4E-BP1 (38), PCDC4 (39–41)
Prostate	hnRNP C (42), YB-1 (43), eIF4E (44)
Gynecologic malignancies	YB-1 (45), HuR (46, 47), IGF2BP1 (48), IGF2BP3 (49, 50), 4E-BP1 (51), PCDC4 (52)
Central nervous system	YB-1 (53), IGF2BP1 (54), PCDC4 (55), eEF2 kinase (eEF2K) (56), TRM6/61 (57)

## Overview of the Eukaryotic Translation

The process of translation can be divided into four major phases: initiation, elongation, termination, and ribosome recycling.

During the initiation phase, a preassembled 43S preinitiation complex is recruited to the m^7^Gppp capped 5′ end of the mRNA (2). The 43S preinitiation complex is formed by the association of the 40S ribosomal subunit with the eukaryotic initiation factors (eIF) 1, 1A, 2, 3, 5, and the ternary complex (composed of eIF2, initiator Met-tRNA_i_, and GTP). Preinitiation complexes attach to 5′ capped untranslated region (UTR) with the cooperative action of eIF4F and eIF4B (58).

eIF4F is composed of the DEAD-box RNA helicase eIF4A, the 5′ cap-binding protein eIF4E, and the scaffolding protein eIF4G (58). eIF4A in mammals exists two highly related isoforms [eIF4AI and eIF4AII; a third isoform, eIF4AIII, acts as a translation initiation factor specifically for the nuclear cap-binding complex bound mRNAs and efficiently unwinds secondary structures in their respective 5′ UTRs (59)] which, despite being interchangeable in the eIF4F complex and sharing 90% homology (60), they seem to have some important functional distinctions (61). The activity of eIF4A is modulated by two homologous RNA-binding proteins, eIF4B and eIF4H (60, 62). eIF4B and eIF4H stimulate the helicase activity of eIF4A, allowing the latter to unwind longer and more stable 5′ UTR structures (63). They also stabilize single-stranded unwound 5′ UTR regions and prevent reannealing. In doing so, they also promote unidirectional 5′ to 3′ ribosome scanning (62). eIF4B and eIF4H share a common binding site on eIF4A and, accordingly, their interactions with eIF4A are mutually exclusive (64). Furthermore, eIF4F-bound eIF4E stimulates the helicase activity of eIF4A independent of eIF4E’s 5′ cap-binding function (65). Last, eIF4G also interacts with the poly(A)-binding protein (PABP); PABP associates with the mRNA 3′ poly(A) tail to circularize and stabilize the mRNA (66). The eIF4G–PABP interaction is not absolutely required for ribosome recruitment but enhances translational efficiency (67).

Besides eIF4E and eIF4F, other cap-binding proteins and complexes have been described (68, 69) that drive selective translation. eIF4E2 is a cap-binding homolog of eIF4E which mediates the selective translation of transcripts involved in the adaptive hypoxic response (69). Under hypoxic conditions, eIF4E is sequestered by hypophosphorylated 4E binding protein (4E-BP) (see Regulation of eIF4F Complex Assembly and Activity) while eIF4E2 is relatively spared; the RNA-binding protein RBM4 recruits hypoxia-inducible factor 2α (HIF2α) to a specific RNA element (RNA hypoxia response element) in the 3′ UTR of select mRNAs and this complex in turn diverts translation of the respective RNAs through the cap-bound eIF4E2 (69). eIF4E2-mediated translation has been shown to be critical for tumor growth in sizes greater than the oxygen diffusion limit (70). Oxygen tension as well as development and differentiation status seems to determine the switch between eIF4E2- and eIF4E-mediated modes of cap-dependent translation initiation (71). Last, eIF3-specialized translation is a recently described mode of translation initiation that involves 5′ cap recognition by eIF3d (68, 72). An internal stem–loop structure in the 5′ UTR is also concurrently required for eIF3 recognition and recruitment (68, 72). The genes that are amenable to this mode of translation have been identified using photoactivatable ribonucleoside-enhanced crosslinking and immunoprecipitation and gene ontology analysis revealed that they are functionally enriched in cancer-associated cell growth regulatory pathways, including apoptosis, cell cycling, and differentiation (72). Examples of these genes include *c-Jun, calcineurin B, CDK12*, and *BTG1*. Interestingly, eIF3 has opposing translational regulatory functions: it promotes the translation of *c-JUN*, which is a proto-oncogene; while it blocks the translation of *BTG1*, whose overexpression impairs invasive growth in human lung cancer cells (72). The factors that determine the transcript-specific translational modulatory function of eIF3 is an open question. Intriguingly, a *cis*-acting RNA element has been identified in the *c-Jun* 5′ UTR that blocks eIF4F even when the 5′ cap is intact and the internal stem-loop eIF3-recognition site is deleted (68). Such elements that block or render the association of the eIF4F with the 5′ cap inefficient are being increasingly recognized and their role is to direct mRNAs into a specific translation pathway (72) [see below, translation inhibitory elements (TIEs)].

The preinitiation complex scans downstream the 5′ UTR, inspecting successive triplets as they enter the P(peptidyl) decoding site of the ribosome for complementarity to the anticodon of Met-tRNA_i_ (2). Hydrolysis of eIF2-bound GTP is stimulated by eIF5 in the scanning preinitiation complex, but completion of the reaction is impeded at non-AUG triplets (73). A perfect match with an AUG start codon triggers the arrest of scanning and the irreversible hydrolysis of the GTP in the ternary complex (2). Start codon recognition leads to the release of the initiation factors and the joining of the large (60S) ribosomal subunit to form the 80S initiation complex. The 80S complex is then ready to accept the appropriate aminoacyl-tRNA into the A (aminoacyl) site and synthesize the first peptide bond (2).

Translation of some eukaryotic mRNAs can be initiated independently of the m^7^Gppp cap by recruitment of the 40S ribosomal subunit to a *cis-*acting element located in the 5′ UTR called internal ribosome entry site (IRES). IRES activity is modulated (usually enhanced) by proteins called IRES *trans-*acting factors (ITAFs) (74). IRESs are structurally and functionally diverse (75). There is no uniformity in the way they operate as well as their factor requirements. Variable interactions with canonical initiation factors, ITAFs, and the 40S ribosome are thought to lead to proper positioning of the initiation codon to the ribosomal P-site (76). For some IRESs, the recruitment of the 40 S ribosome involves mRNA–ribosome RNA (rRNA) base pairing between the IRES and 18S rRNA (77, 78). In addition, TIEs in the 5′ UTR may block cap-dependent translation and divert the translation of the respective transcripts through the IRES (79). rRNA methylation and pseudouridylation enhance IRES-mediated translation (18, 80, 81); these covalent modifications are mediated by the enzymes fibrillarin (FBL) (18) and dyskerin (DKC1) (81), respectively. Also, ribosomal proteins ribosomal protein L38 (79) and ribosomal protein S25 (82) have been shown to be important for IRES-mediated translation. These covalent modifications and ribosomal protein associations modulate the mRNA affinities and IRES-translational capabilities of the ribosomes. IRES-mediated translation is frequently upregulated during stress conditions when cap-dependent translation initiation is compromised (83). However, for some transcripts containing a TIE such as the Hox mRNAs, IRES-mediated translation may constitute the only mode of translation initiation (79). An increasing number of genes with unequivocal relevance to cancer biology have been found to contain IRES; examples include *VEGF* (84), *Bcl-2* (85), *FGF1* (86), *c-Jun* (87), *Aurora A kinase* (88), *c-myc* (89, 90), *XIAP*, and *IGF1R* (78, 91). To that end, the expression of ITAFs that modulate the translation of these mRNAs has been investigated and correlated with clinicopathologic parameters and outcomes and may serve as a biomarker in specific cancers (Table 1; Table S1 in Supplementary Material). Although studies have shown that typically IRES-mediated translation is upregulated under cancer-relevant stress conditions, the overall role of this non-canonical mode of translation initiation seems to be more complex, as transcripts encoding proapoptotic proteins such as the apoptotic protease activating factor (APAF1) also contain IRESs (92). Changes in the abundance or activity of the ITAFs (83) and alterations in the composition of specific ribosome subpopulations may modulate IRES-mediated translation.

Translation elongation is mediated by elongation factors eEF1 and eEF2 (93). eEF1A in complex with GTP binds to and delivers aminoacylated tRNA to the A-site of the ribosome. GTP is hydrolyzed when codon-anticodon recognition occurs; the eEF1A-guanosine-5’-diphosphate (GDP) then exits the ribosome and is recycled to eEF1A-GTP by the nucleotide exchange factor eEF1B complex (94). eEF2 mediates the translocation of the nascent protein chain from the A-site to the P-site of the ribosome. eEF2 can be phosphorylated and inactivated by its cognate kinase eEF2 kinase (eEF2K), resulting in deceleration of translation elongation (95). eEF2K in turn is regulated by a diverse array of inputs including the mTORcomplex 1 (mTORC1) pathway among other nutrient-sensing and growth factor activated signaling pathways. Ribosome profiling has shown that global translation elongation rates are remarkably consistent across diverse classes of transcripts (96). However, at a single mRNA level, ribosomes move in a stop-and-go manner and can pause at various consensus sites which, remarkably, do not correspond to rare codons where tRNA recruitment might be expected to be rate limiting (96).

Translation termination in eukaryotes occurs in response to stop codons in the ribosomal A-site and it involves the concerted action of two eukaryotic release factors eRF1 and eRF3 (97, 98). eRF1 is responsible for stop codon recognition and the hydrolysis of peptidyl-tRNA, whereas eRF3 strongly stimulates peptide release by eRF1 in a GTP-dependent manner. After peptide release, eRF1 remains bound to post-termination complexes and, together with the ATP-binding cassette protein ABCE1, dissociates the complex into a 60S subunit and tRNA- and mRNA-associated 40S subunits (99). ABCE1also mediates the recycling of post-termination complexes (100), an essential process in maintaining a pool of free ribosomes in the cell.

In some cases, post-termination complexes do not undergo complete recycling; 40S subunits remain bound to mRNA, and termination is followed by reinitiation, usually downstream of the stop codon (98). Indeed, multiple studies that employed ribosome profiling have revealed translation in the 3′ UTRs (100, 101) originating from reinitiation rather than readthrough of the main open reading frame (ORF) stop codon (100). 3′ UTR translation is upregulated under conditions of nutrient starvation in the yeast (100); other stress conditions that upregulate this pattern of translation and its implications in cancer are an open question. The overwhelming majority of such reinitiation events seems to occur when the 5’-proximal ORF is short and can significantly impact the translation of the downstream protein-coding ORF (98). This is particularly relevant in the human transcriptome where nearly half of all transcripts are at least computationally predicted to contain upstream or overlapping reading frames whose translation in general represses the translation of the downstream coding sequence (102). It is generally thought that reinitiation involves the same factors as standard initiation; however the complex MCT-1/DENR is a non-canonical initiation factor that specifically promotes reinitiation in eukaryotes (103). In fact, certain mRNAs containing upstream ORFs (uORFs) with strong Kozak sequences (i.e., consensus sequences that promote translation) selectively require MCT-1/DENR for the proper translation of the downstream main coding sequence, and interestingly, these mRNAs are enriched for transcriptional regulators and oncogenic kinases (103).

## Translational Regulation and Dysregulation in Cancer

### Transcript-Specific Elements That Modulate Translation

**Figure 1:**
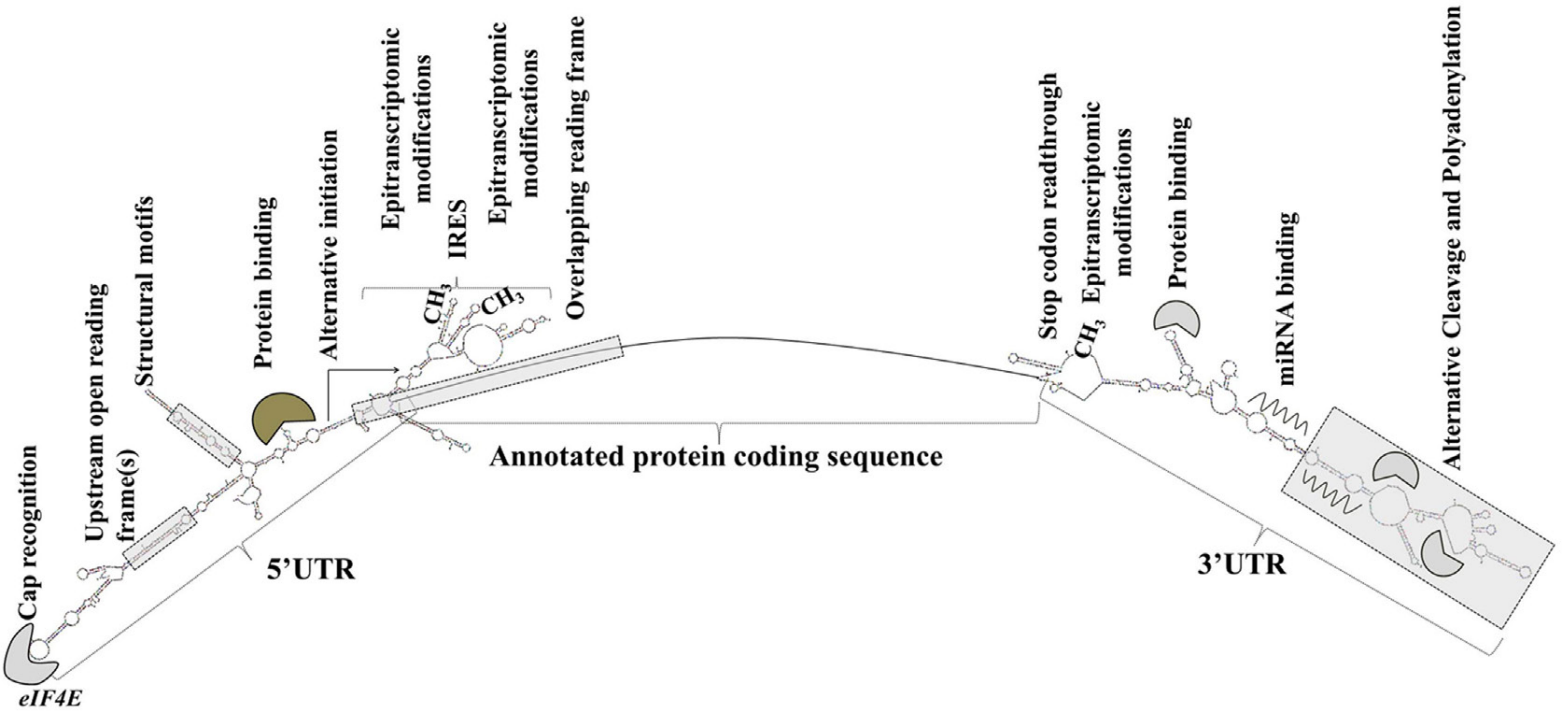
Transcript-specific elements that modulate messenger RNA metabolism, translation initiation, and efficiency. Gene expression is extensively and dynamically modulated at the post-trascriptional level in cell type-specific and context-dependent manner.

Sequence elements and structural features of the individual mRNA transcript allow for the dynamic modulation of its translation (93) (Figure 1).

Elements in the 5′ UTR include cap recognition patterns (discussed below in relation to eIF4E, the principle cap-binding protein), IRESs, uORFs, motifs with RNA-binding protein recognition sequences or specialized secondary structures, and epitranscriptomic modifications. An example of a 5′ UTR structural element with a transcript-specific translational impact is a 12-nucleotide guanine quartet (CGG)_4_ motif that can form RNA G-quadruplex structures (104). The presence of RNA G-quadruplexes renders the translation of the respective transcripts remarkably sensitive to eIF4A inhibition with the investigational compound silvestrol (104). Transcripts of many oncogenes, superenhancer-associated transcription factors, and epigenetic regulators are eIF4A-dependent and, accordingly, silvestrol-sensitive (104).

3′ UTRs are typically longer than the 5′ UTRs and are thought to allow for greater transcript-selective translational regulation (105). The 3′ UTRs undergo epitranscriptomic modifications and contain miRNA-binding sites; the latter silence gene expression by translational repression and mRNA destabilization (106, 107). Studies have shown that the 3′ UTR constitutes the site where most mRNA–RNA-binding protein interactions occur (108). Through a process called alternative cleavage and polyadenylation (109), cells can modulate the length of the 3′ UTR and consequently, by retaining or excluding miRNA- or RNA protein binding sites, regulate the function, stability, localization and translation efficiency of the respective mRNAs. Cancer cells usurp this mechanism to promote the silencing of antiproliferative genes and evade growth inhibition (109, 110).

#### RNA-Binding Proteins

RNA-binding proteins impact all aspects of RNA biology including transcription, pre-mRNA splicing, polyadenylation, RNA modification, transport, localization, translation, and turnover. Although they bind to RNA through a relatively small repertoire of RNA-binding scaffolds, their affinity and specificities are extensively modulated by auxiliary domains which in turn modulate interactions with other proteins and are subject to posttranslational modifications (111). Although traditionally thought that RNA-binding proteins bind to the 5′ and 3′ UTRs of their target transcripts, the former can also bind, probably to a lesser extent, to the protein-coding region of the message. Depending on the binding site across the target mRNA, RNA-binding proteins can mediate different functions. This has been exemplified with GLD-1 whereby binding to sites across the protein-coding regions of its target transcripts mediated predominantly translational repression (112). RNA-binding proteins with relevance to cancer biology include HuR, the IGF2 mRNA-binding protein (IGF2BP) family (mainly IGF2BP1 and IGFBP3), and cytoplasmic polyadenylation element-binding (CPEB) (113).

HuR (ELAV-like RNA-binding protein 1) stabilizes and/or affects the translation of its target mRNAs by interacting with one or several U- or AU-rich elements (AREs) in their 3′ UTRs (114). Other functions attributed to HuR include involvement in pre-mRNA splicing (115–117) and nuclear export of mRNAs (114). HuR’s function is predominantly regulated by posttranslational modifications which in turn determine HuR’s interactions with its target mRNAs and nucleocytoplasmic shuttling machinery (114). A number of cancer-related transcripts, including mRNAs for proto-oncogenes, cytokines, growth factors, and invasion factors, contain AREs and have been identified as HuR targets. Examples include *c-myc, IGF1R, HIF1*α, *HSP90AB1, eEF2, interleukin 11*, and *CDK6* (116). It has been proposed that HuR has a central tumorigenic activity by enabling multiple cancer phenotypes (118). HuR localizes predominantly in the nucleus where it interacts with introns and is involved in pre-mRNA splicing (115–117). HuR’s cytoplasmic translocation is thought to be the initial and critical step for its target mRNA stabilizing and translational modulatory effects (114, 119, 120). Multiple studies have consistently associated cytoplasmic (but not nuclear) expression with worse clinicopathologic characteristics and outcomes in diverse malignancies (Table 1; Table S1 in Supplementary Material).

The IGF2BPs are oncofetal proteins that are normally expressed only during embryogenesis (121); however, the expression of IGF2BP1 and IGF2BP3 is induced in various malignancies. Examples include neuroblastoma (54) and ovarian carcinoma (48) for IGF2BP1; and triple-negative breast cancer (9), ovarian (49) and endometrial (50) clear cell carcinoma, gastric adenocarcinoma (32), cholangiocarcinoma (33, 122), colon cancer (34, 35), renal cell (123, 124), and urothelial carcinoma (125) for IGF2BP3 (Table 1; Table S1 in Supplementary Material). During development, IGF2BPs are required for proper nerve cell migration and morphological development, presumably by regulating cytoskeletal remodeling and dynamics (126). Likewise, in tumor cells, IGF2BPs modulate cell polarization, adhesion, and migration. Moreover, they are highly associated with cancer metastasis and the expression of oncogenic factors (KRAS proto-oncogene GTPase, MYC, and MDR1) (126). In fact, IGF2BP1 was originally identified as a protein involved in the stabilization of *c-myc* mRNA (127). Furthermore, IGF2BP1 is an ITAF upregulating the IRES-mediated translation of the cellular inhibitor of apoptosis 1 leading to resistance to apoptosis in rhabdomyosarcoma (128). Multiple studies have evaluated the expression of IGF2BP proteins (mainly IGF2BP3) in cancer and have consistently correlated IGF2BP3 expression with the subsequent development of recurrence or metastases in localized cancer and worse clinical outcomes (Table 1; Table S1 in Supplementary Material). There is significant homology among the 3 IGF2BP family members, thus antibodies used to investigate the expression of IGF2BP1 and IGF2BP3 may have not differentiated the paralogs (129).

Last, the CPEB family of RNA-binding proteins (CPEB1–4) bind to cytoplasmic polyadenylation elements (CPEs; consensus sequence UUUUUAU) in the 3′ UTR of target mRNAs (113, 130) and modulate translation (both activation and repression) through regulation of poly(A) tail length (131). Multiple lines of evidence suggest a tumor-suppressive role for CPEB1 (130). The expression level of *CPEB1* mRNA are decreased in a diverse array of malignancies and this reduction has been associated with proliferation, invasion, angiogenesis, increased resistance to nutritional stress, loss of polarity, and epithelial-to-mesenchymal transition (130, 132, 133). CPEB4 on the other hand is overexpressed in pancreatic ductal adenocarcinoma, high-grade gliomas (134), and early in the development of melanoma (135). CPEB4 associates with a large number of CPE-containing mRNAs, which seem to be tissue-specific (134, 135), and steers the translational landscape to support the phenotypic hallmarks of malignancy, i.e., invasive growth (pancreatic adenocarcinoma), uncontrolled tumor growth, and aberrant angiogenesis (high-grade gliomas) (134).

#### Epitranscriptomic Modifications

Although RNA modifications have been known for nearly 60 years, only recently, has it been appreciated how extensive and dynamic these chemical modifications to the mRNA may be (136). Recent technical advances have led to the discovery, identification, and mapping of widespread mRNA modifications with *N*^6^-methyladenosine (m^6^A), 5-methylcytosine (m^5^C), pseudouridine (ψ), *N*^6^,2’-O-dimethyladenosine (m^6^Am), 5-hydroxylmethylcytosine (hm^5^C), inosine (I), and *N*^1^-methyladenosine (m^1^A) (137, 138). Analogous to the epigenetic modifications of the deoxyribonucleic acid (DNA), mRNA modifications involve epitranscriptomic “writers,” “erasers,” and “readers”; i.e., enzymes that insert, remove, or recognize these covalent modifications, respectively, and facilitate mRNA-templated processes. Single nucleotide polymorphisms in the m^6^A eraser alpha-ketoglutarate dependent dioxygenase (*FTO*), a gene that traditionally has been associated with obesity, have been associated with melanoma (139) and estrogen receptor-negative breast cancer (140). These mRNA modifications can potentially affect most posttranscriptional steps in gene expression and, specifically for m^6^A, are highly dynamic (137). Common themes that arise so far link m^6^A modifications with mRNA stability, splicing, and translational efficiency (137). m^6^A sites are enriched near stop codons (141) and an association between m^6^A modification and proximal alternative cleavage and polyadenylation, i.e., 3′ UTR shortening has been established (142). m^6^A modifications in the 5′ UTR have been associated with cap-independent translation (143, 144). The role of the epitranscriptome in cancer remains an open area of investigation.

#### RNA Editing

The most frequent type of RNA editing in the human transcriptome is adenosine-to-inosine (A-to-I), which involves hydrolytic deamination of adenosine by the adenosine deaminase, RNA-specific (ADAR) family of RNA editases, ADAR1, ADAR2/ADARB1, and ADAR3/ADARB2 (145). The resulting inosine bases are subsequently read as guanosines, thus inducing A-to-G posttranscriptional changes. Most RNA editing sites occur in non-coding sequences, such as 5′ UTRs, 3′ UTRs, and intronic sequences (145). ADARs may influence gene expression by modulating mature miRNA biogenesis, splicing, alternative cleavage, and polyadenylation (146). Aberrant activation of ADAR-mediated RNA editing has emerged as a driver of cancer progression (145) but how it distorts the translational landscape is an open question.

### Regulation of eIF4F Complex Assembly and Activity

Oncogenes and oncogenic signaling pathways uniformly converge at the level of translation and the transformative potential of oncogenic insults “bottlenecks” at the level of translation (44, 147, 148): ribosomal protein (147) and eIF4E haploinsufficiency (148) or non-phosphorylatable eIF4E (44) suppress the transformative potential of Myc and Ras oncogenes.

**Figure 2:**
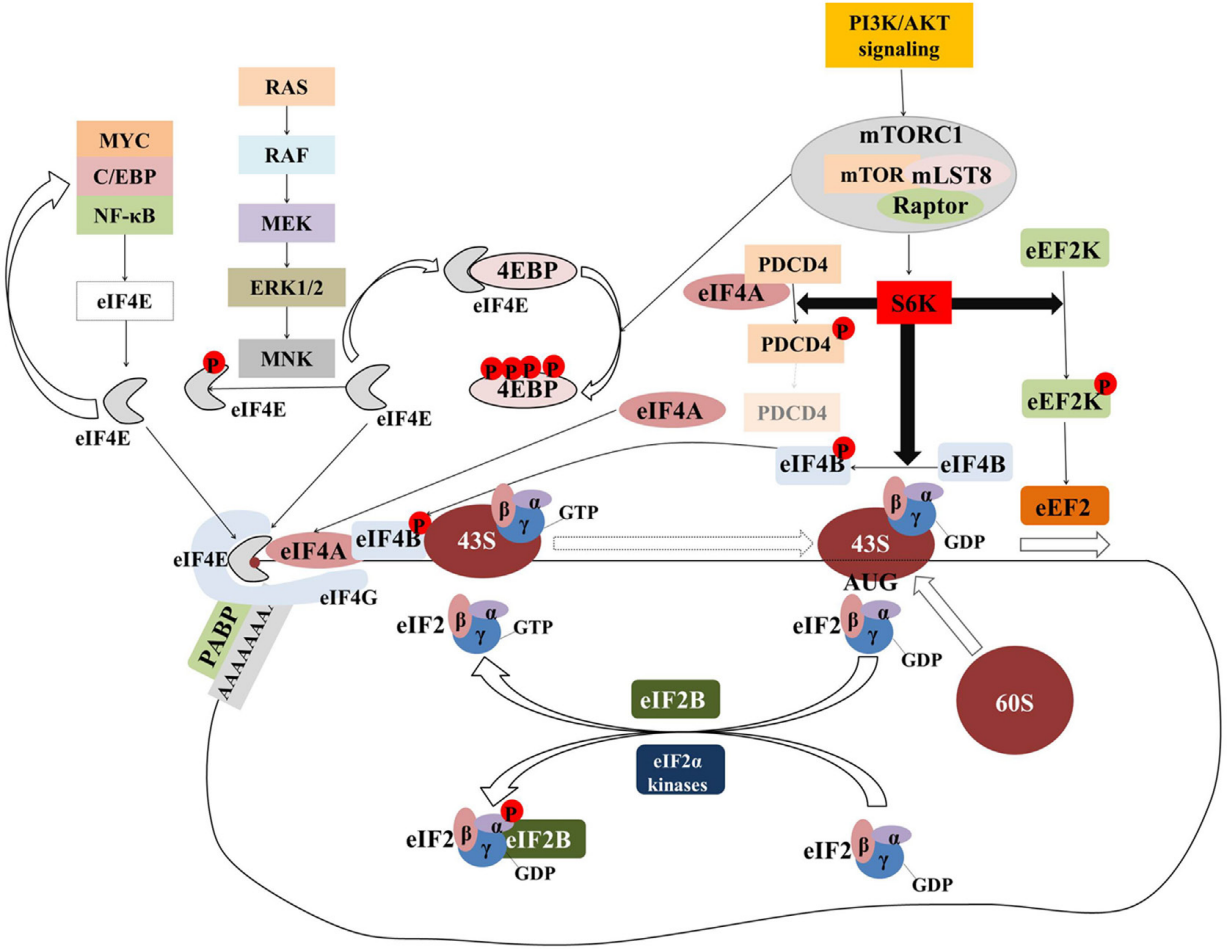
The majority of translational regulation is exerted at the level of initiation. Overview of the regulation of eIF4F and ternary complex assembly and activity. Transcription factors (MYC, C/EBPα, NF-κB) upregulate the transcription of components of the eIF4F complex which in turn upregulates the translation of transcripts encoding for the former generating a “feed-forward” loop. Signaling pathways activate the MNK serine/threonine kinases which in turn phosphorylate eIF4E. Although eIF4E phosphorylation is associated with the acquisition of critical hallmarks of malignancy, the mechanism by which this is achieved is an open question. The mTORcomplex 1 (mTORC1) is a critical nexus linking aberrant signaling with translation. Phosphorylation of the 4E binding proteins (4E-BPs) releases eIF4E to form eIF4F complex. Activation of ribosomal protein S6 kinases (S6Ks) leads to the phosphorylation and subsequent degradation of programmed cell death 4 (PDCD4), which in turn releases eIF4A to form eIF4F complex. At the same time, phosphorylated eIF4B stimulates the helicase activity of eIF4A which is necessary to unwind the highly structured 5′ untranslated regions of many oncogenes. Phosphorylation of eEF2 kinase (eEF2K) leads to its inactivation and accelerated translation elongation by the uninhibited eEF2. Ternary complex formation is regulated by means of eIF2α phosphorylation. eIF2B catalyzes the guanosine-5′-diphosphate (GDP)-to-GTP exchange to allow another cycle of translation initiation to occur. In response to stress, eIF2α is phosphorylated at serine 51 and becomes a competitive inhibitor of eIF2B. Poly(A)-binding protein (PABP) associates with the messenger RNA (mRNA) 3′ poly(A) tail to circularize and stabilize the mRNA and bolster its translation.

Initiation has been considered the rate-limiting step in translation and has been intensively investigated. Figure 2 provides an overview of the components and regulation of translation initiation. In many human cancers, eIFs are either overexpressed or ectopically activated by oncogenic signaling cascades, resulting in increased survival and accelerated proliferation (149). To that end, many eIFs are considered *bona fide* proto-oncogenes (149). Multiple studies in diverse malignancies and settings have investigated correlations between expression levels of eIFs with clinical parameters or outcomes (Table 1; Table S1 in Supplementary Material). Components of the eIF4F complex are perhaps the most intensively investigated factors with potential utility as biomarkers. In the sections that follow, we outline the pathobiology of eIF4F and its components which underpins its potential utility as a biomarker.

#### The Oncogenic Transformative Potential of eIF4E

eIF4E was the first translation initiation factor documented to have oncogenic transformative potential (150, 151). Although eIF4E is the least abundant (i.e., most limiting) of the initiation factors (60), multiple studies have shown that partial depletion of eIF4E has only a moderate impact on protein synthesis rates (152). Indeed, mice haploinsufficient in *Eif4e* develop normally with near normal global mRNA translation levels (148). This *Eif4e* excess becomes critical in the context of oncogenic signaling (148). Although eIF4E is required for cap-dependent translation of all mRNAs, changes in its levels have a highly selective (as opposed to global) impact on a subset of mRNAs that encode proteins with prosurvival and proliferative functions. Transformed cells usurp this excess *Eif4e* to translate genes involved in cell signaling, apoptosis, ribosome biogenesis, control of proteasome activity, nucleotide biosynthesis, oxidative phosphorylation, and the oxidative stress response, all of which act in concert to promote tumorigenesis (148). Many of these eIF4E-responsive mRNAs possess complex and highly structured 5′ UTRs (153) that impede ribosome recruitment and scanning; eIF4E availability allows the cell to overcome this impediment by virtue of stimulating eIF4A helicase activity (65). The basis of eIF4E translational selectivity may also rely on the fact that, although m^7^Gppp is the invariant component of the 5′ cap, there is variability in the affinity with which eIF4E associates with the 5′ cap determined by the second nucleotide (67, 154). Of note, the first and second nucleotide following the m^7^Gppp can be variably methylated; the physiologic significance of these modifications is not yet known. Moreover, the association of the eIF4E with the cap blocks decapping leading to mRNA stabilization (67). Besides its role in translation, eIF4E mediates nucleocytoplasmic transport of specific transcripts such as cyclin D1 which promotes cell cycle progression, and this function contributes to the transformative activity of eIF4E (155).

A similarly selective impact on translation has been observed with eIF4E phosphorylation. eIF4E is phosphorylated by the MNK1/2 serine/threonine kinases, which are activated in response to mitogenic and stress signaling downstream of ERK1/2 and p38 MAP kinase, respectively (Figure 2). eIF4E phosphorylation [which is dispensable for normal growth and development and, *per se*, does not globally affect protein synthesis (156)] is associated with upregulated translation of a select subset of protumorigenic mRNAs (44). The mechanistic basis by which phosphorylated eIF4E achieves that remains a critical unanswered question; in fact, in a seemingly counterintuitive fashion, phosphorylated eIF4E has lower affinity for the cap structure (60, 157, 158). Nonetheless, eIF4E phosphorylation underpins epithelial-to-mesenchymal transition, invasion, and migration (159, 160) and is associated with higher-grade and hormone-refractory prostate cancer (44).

#### Signaling Pathways Upregulate eIF4F Assembly and Activity through Mechanistic or Mammalian Target of Rapamycin (mTOR)

The mammalian/mechanistic target of rapamycin is a serine/threonine kinase that forms 2 distinct multiprotein complexes termed mTOR complex 1 and 2 (Figure 2). Each complex has distinct substrates and, accordingly, regulates different cellular processes (161). mTORC1 links many extracellular and intracellular nutrient and growth cues to the translation process mainly by regulating the eIF4F assembly (66). Simultaneously, mTORC1 (in association with CK2) regulates ternary complex recycling to couple coordinate changes in eIF4F assembly and, consequently modulate the rate-limiting step of translation initiation (162).

mTORcomplex 1 stimulates global protein synthesis, as well as translation of a specific subset of mRNAs (163). Eukaryotic translation initiation factor 4E-BPs and ribosomal protein S6 kinases (S6Ks) are the most extensively studied and best-understood downstream effectors of mTORC1 (163).

4E binding proteins modulate eIF4F assembly by competing with eIF4G for the same binding site on eIF4E. 4E-BPs’ affinity for eIF4E depends on the phosphorylation status of the former, which in turn depends on the activity of mTORC1. Activation of the mTORC1 pathway leads to the phosphorylation of 4E-BPs, which in turn dissociate from eIF4E allowing the latter to associate with eIF4G and form the eIF4F complex. In mammals, there are three known 4E-BPs (4E-BP1, 4E-BP2, and 4E-BP3). The activity of 4E-BP1 and 4E-BP2 is primarily controlled by phosphorylation. mTOR inhibitors downregulate 4E-BP1 and 4E-BP2 phosphorylation leading to eIF4E sequestration. However, a common mechanism of acquired resistance to mTOR inhibitors involves downregulation of 4E-BP1 and 4E-BP2, which leads to increased eIF4E availability. The eIF4E:4E-BP ratio has been proposed as a predictive biomarker of response to mTOR inhibitors that can personalize treatment selection (164). Unlike 4E-BP1 and 4E-BP2, 4E-BP3 is mainly regulated by transcriptional induction (165). 4E-BP3 is an important determinant in mediating the antiproliferative effects of mTOR inhibitors and induction of its expression has been associated with antitumor response to mTOR inhibition (165). 4E-BP3 induction of expression upon mTOR inhibition can potentially predict response as well as duration of response associated with mTOR inhibition.

Ribosomal protein S6 kinases (S6K1 and S6K2 in mammals) have various downstream substrates including ribosomal protein S6, eIF4B, and programmed cell death 4 (PDCD4). The impact of ribosomal protein S6 phosphorylation on translation is not very well understood. eIF4B, however, is an important auxiliary factor that stimulates the helicase activity of eIF4A; its phosphorylation by S6K (166) as well as other kinases (167) selectively and positively impacts the translation of protumorigenic transcripts with structured 5′ UTRs (168). The phosphorylation of PDCD4 by S6Ks leads to its proteasomal degradation and the liberation of eIF4A from PDCD4-eIF4A inhibitory complexes (169). Alongside upregulating translation initiation, S6Ks also phosphorylate eEF2K which in turn, accelerates elongation. Last, mTORC1 upregulates ribosome biogenesis by activating RNA polymerase I transcription initiation factor TIF-1A (170), and tRNA synthesis by phosphorylating and suppressing the RNA polymerase III inhibitor MAF1 (171, 172) (see tRNA Abundance and Modifications Fostering Oncogenic Translation).

#### Oncogenic Transcription Factors Upregulate the eIF4F Complex

The *c-myc* oncogene has a pervasive impact on translation and the upregulation of translational output is a critical determinant of its oncogenic activity *in vivo* (147). Myc modulates the transcription of 10–15% of all genes and Myc-target genes belong to diverse functional categories (173); specifically regarding translation, Myc upregulates ribosome biogenesis, tRNA levels, and key translation initiation and elongation factors (174). All components of the eIF4F complex (eIF4E, eIF4AI, and eIF4GI) are under the direct transcriptional control of c-Myc and are coordinately upregulated when *c-myc* is overexpressed (174, 175). Increased eIF4F, in turn, selectively upregulates the translation of protumorigenic mRNAs including c-Myc mRNA (Figure 2). Normally, the activity of this c-Myc–eIF4F feed-forward loop is modulated by negative regulators of Myc or downregulators of eIF4F assembly [e.g., mTORC1, see Signaling Pathways Upregulate eIF4F Assembly and Activity through Mechanistic or Mammalian Target of Rapamycin (mTOR)]. In cancer, however, the c-Myc–eIF4F feed-forward loop fuels neoplastic progression as the negative checkpoints of this loop are circumvented by mutations or perturbations in signaling pathways (174).

Other transcription factors can also modulate eIF4F assembly and activity (66). In a positive feedback loop, the leukemogenic p30 isoform of C/EBPα upregulates the transcription of eIF4E and eIF4E in turn upregulates the translation of C/EBPα (176). The dysregulated activity of this loop may underpin the dysplastic phenotype of myelodysplastic syndrome/acute myelogenous leukemia (AML) associated with nucleophosmin deficiency (176). In the context of M4 and M5 AML, NF-κB directly upregulates the transcription of eIF4E; this association has not been seen in other AML subtypes or normal hematopoietic cells (177) suggesting that other transcription factors besides c-Myc may upregulate eIF4E in a context-dependent or tissue-specific manner. Under conditions of hypoxia in breast cancer cells, HIF1α promotes eIF4E1 expression acting through hypoxia response elements in the proximal promoter region of *eIF4E1* (178). In the context of this adaptive response, eIF4E1 upregulates the translation of a select subset of mRNAs important for mammosphere formation and growth (178).

#### Posttranscriptional Upregulation of eIF4E in Cancer

The eIF4E mRNA stability is modulated by 2 competing 3′ UTR RNA-binding proteins, HuR (see RNA-Binding Proteins) and AU-rich binding factor 1 (AUF1/HNRNPD) (179). In cancer, overexpression of HuR leads to eIF4E mRNA stabilization and consequently elevated eIF4E protein levels. By increasing the stability of multiple client transcripts and through the upregulation of translation of a select subset of protumorigenic mRNAs, HuR and eIF4E, respectively, coordinately dysregulate gene expression at the posttranscriptional and translational level (179).

### Regulation of Ternary Complex Formation

eIF2 consists of an α, β, and γ subunit and cycles between a GDP- and GTP-bound forms. Following start codon recognition, eIF2-bound GTP hydrolysis is completed resulting in the formation of eIF2–GDP complex, which in turn dissociates from the 40S ribosomal subunit (180). For another cycle of initiation to occur, GDP bound to eIF2 is replaced by GTP by the guanine exchange factor eIF2B (180). In response to virtually all stresses, eIF2α is phosphorylated at serine 51 resulting in the conversion of eIF2α from a substrate to a competitive inhibitor of eIF2B (180, 181) (Figure 2). Four eIF2α kinases catalyze this phosphorylation in a cell type- and stress-specific manner: protein kinase RNA-activated (PKR) is activated by viral infection, PKR-like endoplasmic reticulum kinase (PERK) is activated by the accumulation of unfolded polypeptides in the lumen of the endoplasmic reticulum, general control non-derepressible 2 kinase (GCN2) is activated by amino acid starvation and ultraviolet (UV) light, and heme-regulated eIF2α kinase (HRI) is activated by heme deficiency and redox stress. Since eIF2B is present in limiting concentrations, phosphorylation of even a small fraction of eIF2α significantly inhibits eIF2–GDP–eIF2–GTP recycling leading to a global inhibition of protein synthesis (181). Nonetheless, the phosphorylation of eIF2α enhances the translation of a select group of mRNAs, which encode for proteins involved in stress adaptation and recovery (181).

The mechanism by which the eIF2α phosphorylation status modulates translation of a given mRNA relies on the presence or absence of uORFs. This has been exemplified by the modulation of the expression of the transcription factor *ATF4* (182). The mouse activating transcription factor 4 (ATF4) mRNA has two uORFs. When eIF2-GTP is abundant in non-stressed cells, ATF4 expression is downregulated as ribosomes scanning downstream of uORF1 reinitiate at the next coding region, uORF2, which overlaps with the *ATF4-*coding sequence. During stress conditions, phosphorylation of eIF2 and the accompanying reduction in the eIF2-GTP levels increase the time required for the scanning ribosomes to become competent to reinitiate translation. This delayed reinitiation allows for ribosomes to scan through the overlapping uORF2 and instead reinitiate at the *ATF4*-coding region. Physiologically, the increased expression of ATF4 contributes to the expression of genes involved in remediation of cellular stress damage (182). Along these lines, polymorphisms in the 5′ UTR that create or delete such uORFs may modulate the translation of the downstream coding sequences under conditions of stress. One such example involves a polymorphism in the 5′ UTR of excision repair 5 endonuclease (ERCC5) present in 35% of Caucasians (183). ERCC5 encodes for a protein directly involved in nucleotide excision repair, i.e., the DNA damage repair pathway that removes bulky DNA adducts induced by exposure to UV radiation and cisplatin. This common polymorphism results in the generation of an uORF in the 5′ UTR of ERCC5 mRNA which in turn is associated with upregulated *ERCC5* expression following DNA damage. At the clinical level, this polymorphism has been associated with significantly lower progression free survival in pediatric patients with ependymoma treated with cisplatin-containing regimens (183).

Additionally, under conditions of stress when eIF2α phosphorylation diminishes significantly the availability of ternary complex, IRES-mediated translation (which does not require eIF2α) is upregulated (181). Under such conditions, Met-tRNA_i_ may form an alternative ternary complex with factors like eIF5B, MCT-1, and ligatin (eIF2D) (181). Through this mechanism, cancer cells can circumvent the global downregulation of protein synthesis mediated by eIF2α phosphorylation and gain a survival advantage by the preferential translation of IRES-containing mRNAs. Transcript isoforms may contain or exclude an IRES as a result of alternative splicing and polymorphisms in the 5′ UTR may have functional implications in the cap-independent translation of the specific transcript. Indeed, two prevalent polymorphisms in the insulin like growth factor 1 receptor (IGF1R) 5′ UTR and more specifically the Loop3 poly(U)-tract of its IRES have functional implications in terms of translation initiation mediated through the IRES; minimizing the length of this poly(U)-tract has been associated with a consistent increase in the activity of the IRES (78). Collectively, eIF2α phosphorylation promotes the translation of select mRNAs that are inefficiently translated in the absence of stress (180). This shift in the translational landscape may explain why elevated levels of eIF2α phosphorylation correlate with cancer cell survival (181).

### Regulation of Translation Elongation

Given its complexity and the requirement for the coordinate function of multiple factors, initiation has been traditionally thought as the rate-limiting step in translation. However, accumulating evidence suggests that translation can also be modulated at the level of elongation (184).

Multiple factors bind to the ribosomal A site during translation elongation and modulate translocation to the endoplasmic reticulum, polypeptide release, ribosome recycling, and mRNA decay (184). Ribosomes stall when the signal recognition particle (SRP) binds the N-terminus of the nascent polypeptide and docks into the ribosomal A site. Further tRNA entry is blocked and elongation is arrested until the ribosome/mRNA complex translocate to the endoplasmic reticulum (184). The SRP pathway is the best understood and thoroughly investigated pathway that regulates spatial organization and compartmentalization of translation (185) but other mechanisms also exist (186). Of note, recent studies have challenged the notion that only secretory proteins are translated in the endoplasmic reticulum; mRNAs encoding both cytosolic and topogenic signal-encoding proteins can be translated in the endoplasmic reticulum with similar translational efficiencies, which are consistently higher than the translational efficiencies in the cytosolic compartment (187).

Translation elongation may be modulated by factors acting on elements in the 3′ UTR. This is exemplified by hnRNP E1 which binds a 33-nucleotide TGF-B-activated translation (BAT) element (188, 189). This element is present on the 3′ UTR of transcripts that mediate epithelial–mesenchymal transition (EMT). Through this interaction, hnRNP E1 stalls translation elongation by inhibiting the release of eEF1A1 from the ribosomal A site. TGF-β-mediated hnRNP E1 phosphorylation, however, disrupts the BAT complex, thereby restoring translation elongation of the respective EMT-promoting transcripts (188, 189).

Translation elongation is also regulated by the phosphorylation status of eEF2 (190). During the stage of tumorigenesis, cells in whom oncogenic signaling pathways have been activated may rely on uninhibited translational elongation for malignant transformation (191). In fact, it is the enhanced translational elongation (rather than enhanced translation initiation) mediated by downregulation of the eEF2K and, consequently, activation of eEF2 that may underpin the increased protein synthesis during the stage of tumorigenesis (191). There have been contrasting reports about the role of eEF2K in cancer biology (95, 191, 192). By virtue of downregulating protein synthesis, high levels of eEF2K would be expected to impair proliferation. Indeed, the activity of eEF2K is inhibited by multiple oncogenic signals; however, high levels of eEF2K have been shown to increase the adaptability of tumor cells in nutrient deprivation accounting for the observation that elevated eEF2K transcript levels are associated with poor prognosis in medulloblastoma and glioblastoma multiforme (56) (Table 1; Table S1 in Supplementary Material).

### rRNA Modifications Associated With Cancer: The Oncogenic Ribosome

Recent studies have challenged the view that ribosomes are constitutive components of the translational machinery with no regulatory function (79, 193). There is significant heterogeneity in ribosome composition that results from the differential expression and posttranslational modification of ribosomal proteins, rRNA diversity, and the activity of ribosome-associated factors (193). This ribosomal heterogeneity has a significant impact on the translational output of the transcriptional template (193). As outlined previously, DKC1 and FBL mediate the rRNA pseudouridylation (81) and methylation (18), respectively, which are important ribosomal modifications for IRES-mediated translation (18, 80, 81). Mutations or deletions of the *DKC1* gene encoding DKC1 have a negative impact on the IRES-mediated translation of the tumor suppressor genes *p53* and *p27* predisposing to hematologic and solid malignancies (81). Altered rRNA methylation patterns mediated by FBL are associated with upregulated IRES-mediated translation of many oncogenes including *IGF1R, MYC, FGF1, FGF2*, and *VEGFA* (18). The expression of FBL is regulated by the tumor suppressor p53 and *p53* inactivation, a common genomic alteration that occurs in multiple malignancies, leads to FBL upregulation. High expression of *FBL* mRNA has been associated with poor relapse-free and breast cancer-specific survival (18).

### tRNA Abundance and Modifications Fostering Oncogenic Translation

The human genome contains 61 sense codons, many of which are synonymous on the basis that they encode the same amino acid (194). Synonymous codons are used with variable but non-random frequency across the genome constituting the basis of codon bias (195). Synonymous codons base pair with tRNA isoacceptors, i.e., tRNAs that are charged with the same amino acid but have a unique anticodon sequence. Normally, tRNA transcription via RNA polymerase III is regulated in response to nutrient availability and environmental cues, in coordination with rRNA transcription via RNA polymerase I (196). tRNAs have been typically considered as housekeeping products with little regulatory function; however, misregulation in tRNA abundance and modifications has been inherently linked to human disease including cancer (196).

In cancer, tRNAs are overexpressed to meet the demands of upregulated protein synthesis. Indeed, oncogenes such as *c-myc* and tumor suppressor genes such as *p53* and *RB*, regulate positively and negatively, respectively, the transcription of RNA polymerase III (197). The transcription of RNA polymerase III (and consequently tRNA synthesis) is also modulated by oncogenic signaling pathways; ERK phosphorylates the transcription factor TFIIIB, which in turn upregulates the transcription of RNA polymerase III (198), while mTORC1 phosphorylates and inactivates MAF1, a repressor of RNA polymerase III activity (172).

Multiple lines of evidence suggest that the implications of upregulated tRNA synthesis in cancer go beyond the simple need to meet the demands of increased protein synthesis. Experimentally, overexpression of Met-tRNA_i_ in human breast epithelial cells reprogrammed the tRNA pool and led to increased metabolic activity and proliferation rates (196). Studies show that changes in the tRNA pool and composition are neither uniform nor random; tRNA overexpression is selective and coordinates with the status of the cell to favor a specific translational program (199, 200). Indeed, malignant proliferating cells and non-malignant differentiating cells have distinct tRNA pools whose anticodon composition suits the codon usage signature of proliferation/cancer-related and differentiation-related/housekeeping genes, respectively (199, 200). In this context, assessment of tRNA pools by tRNA microarrays or other methodologies holds promise as a biomarker to determine the natural history of a tumor or malignant potential of a premalignant lesion.

tRNAs also undergo extensive posttranscriptional modifications; these dynamic modifications have been traditionally linked with an adaptive stress response, whereby mRNA translation is rapidly suppressed or altered (201, 202). However, several recent studies link these modifications with cancer. This is exemplified by the role of the TRM6/61 methyltransferase complex, which mediates the methylation of adenosine at position 58 of Met-tRNA_i_ (57). This important modification stabilizes Met-tRNA_i_, which in turn allows for the selective translation of oncogenic transcripts. Increasing levels of TRM6/61 correlate with the transition from grade II or III gliomas to glioblastomas, i.e., tumors of the central nervous system with progressively more aggressive natural history (57). The elongator acetyltransferase complex subunit 3 and CTU1/2 enzymes mediate the posttranscriptional modification of the wobble uridine 34, a highly conserved modification that contributes to translational fidelity (19). Increasing levels of those enzymes correlated with the transition from normal breast tissue to non-invasive and invasive breast cancer. This posttranscriptional modification is important for the translation of an ITAF (DEK proto-oncogene), which in turn upregulates the IRES-mediated translation of the transcription factor lymphoid enhancer binding factor 1 (LEF1) (19); of note, LEF1 is an important effector of the WNT- and TGFβ-signaling pathways, which lead to invasion and metastases. On the other hand, the isopentenyltransferase *TRIT1*, which catalyzes the addition of N^6^-isopentenyladenosine on residue 37 of tRNAs, is a tumor suppressor gene in lung cancer (24).

Aminoacyl-tRNA synthetases catalyze the ligation of amino acids to their cognate tRNAs (203). Some of the synthetases are tightly bound together in a large multisynthetase complex, with three tRNA synthetase associated proteins at the core designated as aminoacyl-tRNA synthetase-interacting multifunctional protein (AIMP) 1, 2, and 3 (203). Eukaryotic synthetases contain unique extensions and domains, which, alongside with variable subcellular localization (nuclear, cytoplasmic, or secreted) either within complexes or free, endows them with an increasingly recognized functional diversity extending beyond translation (203, 204). Although the importance of the canonical functions of the tRNA synthetases in cancer has been recognized early (205, 206), it is the additional non-canonical activities that are more indicative of a role in tumorigenesis (207).

## Concluding Remarks and Future Perspectives

Collectively, over the recent years, the complexity of posttranscriptional regulation of gene expression is becoming increasingly recognized. New technologies and advances in next-generation sequencing have allowed us to dissect the mechanistic underpinnings of translational regulation at an unprecedented scale and resolution (93). Translation becomes distinctively dysregulated in cancer and this dysregulation is critical for oncogenes and oncogenic signaling pathways to carry out their transformative potential while at the same time, endows cancer cells with distinctive adaptive capabilities to a diverse nature of stresses.

In this context, multiple factors involved in the posttranscriptional regulation of gene expression arise as biomarkers with potential diagnostic, prognostic, or predictive utility. Assessment of these factors can help us in the diagnosis of equivocal cases, determine the malignant potential of premalignant lesions, predict response to a specific therapy, determine the risk of recurrence or cancer-related death, and altogether help us in clinical decision making and the refinement of our treatment approaches. Rapidly emerging new data are likely to provide even greater insight and rationale for the utilization of these translational regulatory factors in patient stratification and treatment selection. Moreover, as new drugs targeting the translational machinery enter clinical investigations, the role and importance of those biomarkers are expected to expand.

## Author Contributions

WG conceived the manuscript. CV collected the data and drafted the manuscript. WG and SB critically reviewed the manuscript. All authors approved the final version to be published.

## Conflict of Interest Statement

The authors declare that the research was conducted in the absence of any commercial or financial relationships that could be construed as a potential conflict of interest. The handling editor declared a shared affiliation, though no other collaboration, with the authors and the handling editor states that the process met the standards of a fair and objective review.

## Abbreviations

4E-BP, eIF4E-binding protein; A, adenosine; A-site, acceptor site; ABCE1, ATP-binding cassette subfamily E member 1; ADAR, adenosine deaminase, RNA-specific; ADARB1/2, adenosine deaminase, RNA-specific B1/2; AIMP, aminoacyl-tRNA synthetase-interacting multifunctional protein; AML, acute myelogenous leukemia; APAF1, apoptotic protease activating factor; ARE, AU-rich element; ATF4, activating transcription factor 4; BAT, TGF-B-activated translation element; BRAF, B-Raf proto-oncogene, serine/threonine kinase; Bcl-2, Bcl-2 apoptosis regulator; BTG1, BTG anti-proliferation factor 1; C/EBPα, CCAAT/enhancer binding protein alpha; CDK6, cyclin dependent kinase 6; CDK12, cyclin dependent kinase 12; CPE, cytoplasmic polyadenylation element; CPEB1–4, cytoplasmic polyadenylation element-binding protein 1–4; c-Jun, Jun proto-oncogene; c-myc, v-myc avian myelocytomatosis viral oncogene homolog; CTU1/2, cytosolic thiouridylase subunit 1/2; DEAD, Asp-Glu-Ala-Asp (tetrapeptide shared by the DEAD-box family of proteins); DEK, DEK proto-oncogene; DKC1, dyskerin; DNA, deoxyribonucleic acid; eEF1/2, eukaryotic translation elongation factor 1/2; eEF2K, eEF2 kinase; eIF (1, 1A, 2, 2α, 2B, 2D, 3, 3d, 4A, 4B, 4F, 5, 5B), eukaryotic initiation factor (1, 1A, 2, 2α, 2B, 2D, 3, 3d, 4A, 4B, 4F, 5, 5B); ELP3, elongator acetyltransferase complex subunit 3; EMT, epithelial–mesenchymal transition; ER, estrogen receptor; ERCC5, excision repair 5 endonuclease; eRF1/3, eukaryotic translation termination factor 1/3; ERK1, mitogen-activated protein kinase 3; ERK2, mitogen-activated protein kinase 1; FBL, fibrillarin; FGF1/2, fibroblast growth factor 1/2; FTO, alpha-ketoglutarate-dependent dioxygenase; GCN2, general control non-derepressible 2 kinase; GDP, guanosine-5’-diphosphate; GLD-1, female germline-specific tumor suppressor gld-1; GTP, guanosine-5’-triphosphate; HIF1α/2α, hypoxia-inducible factor 1α/2α; hm^5^C, 5-hydroxylmethylcytosine; hnRNP A1/C, heterogeneous nuclear ribonucleoprotein A1/C; hnRNP E1, poly(rC)-binding protein 1; HRI, heme-regulated eIF2α kinase; HSP90AB1, heat shock protein 90 alpha family class B member 1; HuR, ELAV-like RNA-binding protein 1; I, inosine; IGF1R, insulin-like growth factor 1 receptor; IGF2BP1/3, insulin-like growth factor 2 mRNA-binding protein 1/3; IRES, internal ribosome entry site; ITAF, IRES *trans-*acting factor; LEF1, lymphoid enhancer binding factor 1; m^7^Gppp, 7-methyl guanosine; KRAS, KRAS proto-oncogene GTPase; m^1^A, *N*^1^-methyladenosine; m^5^C, 5-methylcytosine; m^6^A, *N*^6^-methyladenosine; m^6^Am, *N*^6^,2’-O-dimethyladenosine; MAF1, MAF1 homolog, negative regulator of RNA polymerase III; MCT-1/DENR, multiple copies in T-cell lymphoma-1 and density regulated protein; MEK, mitogen-activated protein kinase kinase 7; Met, methionine; Met-tRNA, methionyl tRNA; mRNA, messenger RNA; MDR1, ATP-binding cassette subfamily B member 1; MNK1/2, MAP kinase interacting serine/threonine kinase 1/2; mTOR, mechanistic or mammalian target of rapamycin; mTORC1/2, mTORcomplex 1/2; NF-κB, nuclear factor kappa B; NPM1, nucleophosmin; ORF, open reading frame; p38 MAP, mitogen-activated protein kinase 14; p53, tumor protein p53; PAR-CLIP, photoactivatable ribonucleoside-enhanced crosslinking and immunoprecipitation; P-site, peptidyl site; PABP, poly(A)-binding protein; PDCD4, programmed cell death 4; PERK, PKR-like endoplasmic reticulum kinase; PKR, protein kinase RNA-activated; RB, RB transcriptional corepressor 1; RNA, ribonucleic acid; RPL38, ribosomal protein L38; RPS25, ribosomal protein S25; rRNA, ribosome RNA; S6K, ribosomal protein S6 kinase; SRP, signal recognition particle; TFIIIB, subunit of RNA polymerase III transcription initiation factor IIIB; TGFβ, transforming growth factor beta; TIE, translation inhibitory element; TIF-1A, tripartite motif containing 24; TRIT1, tRNA isopentenyltransferase 1; TRM6/61, tRNA methyltransferase 61A; tRNA, transfer RNA; uORF, upstream ORF; UTR, untranslated region; UV, ultraviolet; VEGF, vascular endothelial growth factor; XIAP, X-linked inhibitor of apoptosis; YB-1, Y-box binding protein 1; ψ, pseudouridine.
