# Supplementary material for: Translational Dysregulation in Cancer: Molecular Insights and Potential Clinical Applications in Biomarker Development
Source: Front Oncol. 2017 Jul 26;7:158. doi: 10.3389/fonc.2017.00158 (PMC5526920; doi:10.3389/fonc.2017.00158)
Supplement: Supplementary file 1 [file Table_1.docx]

**Supplementary table 1. Factors and regulators involved in translation with potential utility as biomarkers**

| Factor | Role/Function | Mode of dysregulation | Clinical Correlates |
| --- | --- | --- | --- |
| IRES *trans-*acting factors | | | |
| hnRNP A1 | ITAF and pre-mRNA splicing factor | Overexpression | High expression and cytoplasmic localization are associated with poor metastatic relapse-free survival in breast cancer(1).  Higher tumor recurrence and shorter survival in hepatocellular carcinoma(2). |
| hnRNP C | ITAF | Overexpression | Poor progression-free and overall survival in gastric cancer(3) |
| YB-1 | ITAF | Expression | Nuclear and cytoplasmic expression correlates with poor prognosis in prostate cancer(4)  Nuclear expression correlates with poor overall survival in osteosarcoma(5) and stage III colorectal cancer(6); poor progression-free and overall survival in uterine cervical carcinoma(7); higher stage, Fuhrman tumor grade, metastasis, and poor overall survival in renal cell carcinoma(8); dedifferentiation, lymphovascular invasion, and unfavorable prognosis in pancreatic adenocarcinoma(9) .  Association with higher risk of relapse and poorer overall survival in breast cancer irrespective of subtype(10).  Increasing expression along the transition from rectitis to tubular adenoma to invasive carcinoma in rectal cancer; association with poorer disease-free and overall survival(11).  Poorer overall survival and high risk of recurrence in small (<2 cm) lung adenocarcinoma(12)  Polymorphism (rs12030724) in *YB-1* affects YB-1 expression and is associated with probability of progression in metastatic prostate cancer(13).  Higher YB-1 levels in the cerebrospinal fluid of patients with Grades III and IV glioma (as compared to patients with Grades I and II glioma). Higher expression with increasing pathologic grade of glioma and association with worse clinical outcome(14).  Higher expression at transcriptional level associated with high-grade sarcomas and worse event-free and overall survival(15) |
| RNA binding proteins | | | |
| HuR | Regulation of mRNA stability | Cytoplasmic overexpression | Characterizes a patient population with resected pancreatic ductal adenocarcinoma that achieved an increased disease-free survival with adjuvant 5FU(16). Patients with low cytoplasmic HuR expression had similarly low disease-free survival with either 5FU or gemcitabine(16).  Association with higher tumor grade and stage, generation of metastases, and decreased disease-specific survival in bladder cancer(17).  Association with poor histologic differentiation, large tumor size, and poor survival in invasive ductal carcinoma of the breast(18). Association of cytoplasmic or nuclear HuR staining with higher grade in breast cancer(19). Association with hormone receptor negativity, p53 positivity, high tumor grade, ductal histology, and decreased survival in non-*BRCA1/2* hereditary breast cancer(20).  Association with higher grade, increased mitotic activity, decreased progression-free and overall survival in ovarian carcinoma(21).  Association with lower overall survival in gastric cancer(22).  Association with larger tumor size, higher stage, and lymphovascular invasion in uterine cervical carcinoma(23). Increase in cytoplasmic expression along the transition from carcinoma *in situ* to microinvasive and invasive squamous cell uterine cervical carcinoma(23).  Association with higher tumor stage in colorectal adenocarcinoma(24).  Association with increased angiogenesis and lymphangiogenesis and worse relapse-free and overall survival in nonsmall cell lung cancer(25). Indirect association (through COX-2 mRNA stabilization) with worse survival in squamous cell carcinoma of the lung(26). |
| IGF2BP1 | Oncofetal RNA binding protein. Also an ITAF for cIAP1(27) | Overexpression | DNA copy number gain and overexpression are associated with higher stage and poorer overall survival in neuroblastoma(28).  Association with higher grade and stage ovarian carcinoma worse recurrence-free and overall survival(29). |
| IGF2BP3 | Oncofetal RNA binding protein | Expression | Association with more aggressive phenotype and decreased overall survival in triple-negative breast cancer(30).  Increased expression in the tumoral myoinvasive front and a trend for decreased relapse-free survival in endometrial clear-cell carcinoma(31).  Association with worse disease-free and overall survival in gastric adenocarcinoma(32). Identification of IGF2BP3 mRNA by RT-PCR in the peritoneal lavage predicts postoperative recurrence and is associated with worse outcome in gastric adenocarcinoma(32).  Decreased disease-specific survival in ovarian clear-cell carcinoma(33).  Correlation with higher tumor grade, tumor stage, lymphovascular invasion, early tumor recurrence , and worse disease-free and overall survival in intrahepatic cholangiocarcinoma(34)  Increased expression distinguishes between high grade dysplasia vs. low grade dysplasia, inflamed and normal tissue in biliary tract. Association with poor overall survival in cholangiocarcinomas(35).  Correlation with higher clinical stage, tumor size, lymph node and distant metastasis in colon cancer(36). Upregulation in colon cancer compared with paired normal colonic mucosa(36). Association with lower disease-free and overall survival in localized colon cancer(36, 37).  Predicts the development of metastases and association with worse overall survival in localized renal cell carcinoma(38, 39)  Predicts the subsequent development of invasive tumors and metastases in superficial invasive urothelial carcinoma; association with worse progression-free and overall survival in superficial and metastatic urothelial carcinoma(40) |
| CPEB4 | RNA binding protein | Overexpression | In gliomas, higher expression correlates with higher grade (associated with a more aggressive natural history) and worse overall survival(41).  Correlation with higher histological grade, lymph node involvement, and worse overall survival in invasive ductal carcinomas of the breast (42).  In colorectal cancer, higher expression is associated with poorer overall survival (43). |
| Translation Initiation | | | |
| eIF4E | 5’ cap binding protein | Overexpression | increased angiogenesis and vascular invasion in breast cancer(44), higher recurrence in triple-negative breast cancer(45), worse outcomes in ER-negative breast cancer(46), higher recurrence and death in lymph node-positive breast cancer(47, 48), independent predictor of recurrence and death in stage I-III breast cancer(49),  steady increase in expression along the transition from benign dysplasia to colorectal adenocarcinoma(50)  increase in expression along the transition from normal prostate tissue, to intraepithelial neoplasia, to hormone-sensitive and to hormone-refractory prostate cancer(51)  worse prognosis in gastric cancer(52),  high local recurrence rate after surgical resection in histologically negative but eIF4E(+) margins in head and neck squamous cell carcinoma(53)  worse failure-free and overall survival in mantle cell lymphoma treated with R hyper-CVAD* (54) |
| eIF4E | 5’ cap binding protein | Phosphorylation | increase in expression along the transition from normal prostate tissue, to intraepithelial neoplasia, to hormone-sensitive and to hormone-refractory prostate cancer(51)  associated with lymph node metastasis and poor prognosis of nasopharyngeal carcinoma(55)  elevated in multiple malignancies and in certain cancers (lung, gastric and colorectal cancers) significantly elevated in early stages(56). |
| 4E-BP1 | Sequestration of eIF4E | Overexpression | Higher recurrence and worse survival in colorectal cancer(57) |
| 4E-BP1 | Sequestration of eIF4E | Phosphorylation | Correlation with poor differentiation, high tumor size, lymph node metastasis, and locoregional recurrence in breast cancer(58)  Independent adverse prognostic factor for progression-free and overall survival in Xp11.2 translocation renal cell carcinoma(59)  Nuclear staining p4E-BP1 is distinctive of endometrial cancer (vs. normal endometrium) and is associated with aggressive tumors with poor prognosis(60) |
| eIF4AI | RNA helicase | Overexpression | Worse outcomes in ER-negative breast cancer(46) |
| eIF4B | Auxiliary factor for eIF4A | Overexpression | Worse outcomes in ER-negative breast cancer(46) |
| eIF4G | Scaffolding protein of the eIF4F complex | Overexpression | Marked overexpression in inflammatory breast cancer as compared to normal mammary tissue(61)  Overexpressed in nasopharyngeal carcinoma as compared to normal tissue; associated with higher stage and worse prognosis in nasopharyngeal carcinoma(62) |
| PDCD4 | Sequestration of eIF4A | Expression | Better outcomes in ER-positive breast cancer(46)  Better prognosis in lung cancer(63)  Better prognosis in patients with high-grade gliomas(64)  Differentiates normal esophageal mucosa (expressed) from cancer (low or no expression); nuclear expression in esophageal cancer is associated with better outcomes(65) |
| PDCD4 | Sequestration of eIF4A | Loss | Progressive loss of PDCD4 along the transition from normal colonic mucosa to adenoma and cancer; associated with poor disease-specific and overall survival(66)  Loss in ovarian cystadenocarcinoma as compared to normal tissue or cystadenoma; worse disease-specific survival(67)  Progressive loss with the transition from Barrett’s mucosa to Barrett’s adenocarcinoma(68)  Association with nodal metastases in oral squamous cell carcinoma(69) |
| eIF2α | Ternary complex | Expression | In conjunction with eIF4E, association with actively proliferating lymphocytes; expression in highly aggressive and aggressive non-Hodgkin lymphomas(70) |
| Re-initiation and IRES-mediated Translation | | | |
| MCT-1 | Forms a complex with DENR to promote re-initiation. Involved in IRES-mediated translation | Overexpression | Association with increased cell proliferation in human primary lymphoid tumors(71) |
| Translation Elongation | | | |
| eEF2K | Deceleration of translation elongation | Overexpression | Poor prognosis in medulloblastoma and glioblastoma multiforme(72) |
| rRNA modifications | | | |
| Dyskerin | rRNA pseudouridylation | Mutation/deletion | Increased incidence of hematologic and solid malignancies(73) |
| Fibrillarin | rRNA methylation | Overexpression | Worse relapse-free and breast cancer-specific survival(74) |
| tRNA modifications | | | |
| TRM6/61 | Met-tRNA_i_ A58 methylation | Overexpression | Increase in expression along the transition from grade II/III gliomas to glioblastomas with progressively more aggressive clinical behavior(75) |
| ELP3 & CTU1/2 | Wobble U34 modification | Overexpression | Increase in expression along the transition from normal breast tissue to non-invasive and invasive breast cancer (76) |
| TRIT1 | Addition of N^6^ isopentenyladenosine on tRNA residue 37 | Decreased expression | Downregulated expression in lung adenocarcinoma(77) |

*****R hyper-CVAD: rituximab plus fractionated cyclophosphamide, vincristine, doxorubicin, and dexamethasone alternating with rituximab plus high-dose methotrexate and cytarabine

1. Cammas A, Lacroix-Triki M, Pierredon S, Le Bras M, Iacovoni JS, Teulade-Fichou MP, et al. hnRNP A1-mediated translational regulation of the G quadruplex-containing RON receptor tyrosine kinase mRNA linked to tumor progression. Oncotarget. 2016;7(13):16793-805.

2. Zhou ZJ, Dai Z, Zhou SL, Fu XT, Zhao YM, Shi YH, et al. Overexpression of HnRNP A1 promotes tumor invasion through regulating CD44v6 and indicates poor prognosis for hepatocellular carcinoma. Int J Cancer. 2013;132(5):1080-9.

3. Huang H, Han Y, Zhang C, Wu J, Feng J, Qu L, et al. HNRNPC as a candidate biomarker for chemoresistance in gastric cancer. Tumour Biol. 2016;37(3):3527-34.

4. Abd El-Maqsoud NM, Osman NA, Abd El-Hamid AM, Fath El-Bab TK, Galal EM. Golgi Phosphoprotein-3 and Y-Box-Binding Protein-1 Are Novel Markers Correlating With Poor Prognosis in Prostate Cancer. Clin Genitourin Cancer. 2016;14(2):e143-52.

5. Fujiwara-Okada Y, Matsumoto Y, Fukushi J, Setsu N, Matsuura S, Kamura S, et al. Y-box binding protein-1 regulates cell proliferation and is associated with clinical outcomes of osteosarcoma. Br J Cancer. 2013;108(4):836-47.

6. Shiraiwa S, Kinugasa T, Kawahara A, Mizobe T, Ohchi T, Yuge K, et al. Nuclear Y-Box-binding Protein-1 Expression Predicts Poor Clinical Outcome in Stage III Colorectal Cancer. Anticancer Res. 2016;36(7):3781-8.

7. Nishio S, Ushijima K, Yamaguchi T, Sasajima Y, Tsuda H, Kasamatsu T, et al. Nuclear Y-box-binding protein-1 is a poor prognostic marker and related to epidermal growth factor receptor in uterine cervical cancer. Gynecol Oncol. 2014;132(3):703-8.

8. Wang Y, Chen Y, Geng H, Qi C, Liu Y, Yue D. Overexpression of YB1 and EZH2 are associated with cancer metastasis and poor prognosis in renal cell carcinomas. Tumour Biol. 2015;36(9):7159-66.

9. Shinkai K, Nakano K, Cui L, Mizuuchi Y, Onishi H, Oda Y, et al. Nuclear expression of Y-box binding protein-1 is associated with poor prognosis in patients with pancreatic cancer and its knockdown inhibits tumor growth and metastasis in mice tumor models. Int J Cancer. 2016;139(2):433-45.

10. Habibi G, Leung S, Law JH, Gelmon K, Masoudi H, Turbin D, et al. Redefining prognostic factors for breast cancer: YB-1 is a stronger predictor of relapse and disease-specific survival than estrogen receptor or HER-2 across all tumor subtypes. Breast Cancer Res. 2008;10(5):R86.

11. Zhang Y, Zhao PW, Feng G, Xie G, Wang AQ, Yang YH, et al. The expression level and prognostic value of Y-box binding protein-1 in rectal cancer. PLoS One. 2015;10(3):e0119385.

12. Zhao S, Guo W, Li J, Yu W, Guo T, Deng W, et al. High expression of Y-box-binding protein 1 correlates with poor prognosis and early recurrence in patients with small invasive lung adenocarcinoma. Onco Targets Ther. 2016;9:2683-92.

13. Shiota M, Fujimoto N, Imada K, Yokomizo A, Itsumi M, Takeuchi A, et al. Potential Role for YB-1 in Castration-Resistant Prostate Cancer and Resistance to Enzalutamide Through the Androgen Receptor V7. J Natl Cancer Inst. 2016;108(7).

14. Zheng J, Dong W, Zhang J, Li G, Gong H. YB-1, a new biomarker of glioma progression, is associated with the prognosis of glioma patients. Acta Biochim Biophys Sin (Shanghai). 2016;48(4):318-25.

15. El-Naggar AM, Veinotte CJ, Cheng H, Grunewald TG, Negri GL, Somasekharan SP, et al. Translational Activation of HIF1alpha by YB-1 Promotes Sarcoma Metastasis. Cancer Cell. 2015;27(5):682-97.

16. Tatarian T, Jiang W, Leiby BE, Grigoli A, Jimbo M, Dabbish N, et al. Cytoplasmic HuR Status Predicts Disease-free Survival in Resected Pancreatic Cancer: A Post-hoc Analysis From the International Phase III ESPAC-3 Clinical Trial. Ann Surg. 2016.

17. Miyata Y, Watanabe S, Sagara Y, Mitsunari K, Matsuo T, Ohba K, et al. High expression of HuR in cytoplasm, but not nuclei, is associated with malignant aggressiveness and prognosis in bladder cancer. PLoS One. 2013;8(3):e59095.

18. Heinonen M, Bono P, Narko K, Chang SH, Lundin J, Joensuu H, et al. Cytoplasmic HuR expression is a prognostic factor in invasive ductal breast carcinoma. Cancer Res. 2005;65(6):2157-61.

19. Denkert C, Weichert W, Winzer KJ, Muller BM, Noske A, Niesporek S, et al. Expression of the ELAV-like protein HuR is associated with higher tumor grade and increased cyclooxygenase-2 expression in human breast carcinoma. Clin Cancer Res. 2004;10(16):5580-6.

20. Heinonen M, Fagerholm R, Aaltonen K, Kilpivaara O, Aittomaki K, Blomqvist C, et al. Prognostic role of HuR in hereditary breast cancer. Clin Cancer Res. 2007;13(23):6959-63.

21. Denkert C, Weichert W, Pest S, Koch I, Licht D, Kobel M, et al. Overexpression of the embryonic-lethal abnormal vision-like protein HuR in ovarian carcinoma is a prognostic factor and is associated with increased cyclooxygenase 2 expression. Cancer Res. 2004;64(1):189-95.

22. Mrena J, Wiksten JP, Thiel A, Kokkola A, Pohjola L, Lundin J, et al. Cyclooxygenase-2 is an independent prognostic factor in gastric cancer and its expression is regulated by the messenger RNA stability factor HuR. Clin Cancer Res. 2005;11(20):7362-8.

23. Lim SJ, Kim HJ, Kim JY, Park K, Lee CM. Expression of HuR is associated with increased cyclooxygenase-2 expression in uterine cervical carcinoma. Int J Gynecol Pathol. 2007;26(3):229-34.

24. Denkert C, Koch I, von Keyserlingk N, Noske A, Niesporek S, Dietel M, et al. Expression of the ELAV-like protein HuR in human colon cancer: association with tumor stage and cyclooxygenase-2. Mod Pathol. 2006;19(9):1261-9.

25. Wang J, Wang B, Bi J, Zhang C. Cytoplasmic HuR expression correlates with angiogenesis, lymphangiogenesis, and poor outcome in lung cancer. Med Oncol. 2011;28 Suppl 1:S577-85.

26. Kim GY, Lim SJ, Kim YW. Expression of HuR, COX-2, and survivin in lung cancers; cytoplasmic HuR stabilizes cyclooxygenase-2 in squamous cell carcinomas. Mod Pathol. 2011;24(10):1336-47.

27. Faye MD, Beug ST, Graber TE, Earl N, Xiang X, Wild B, et al. IGF2BP1 controls cell death and drug resistance in rhabdomyosarcomas by regulating translation of cIAP1. Oncogene. 2015;34(12):1532-41.

28. Bell JL, Turlapati R, Liu T, Schulte JH, Huttelmaier S. IGF2BP1 harbors prognostic significance by gene gain and diverse expression in neuroblastoma. J Clin Oncol. 2015;33(11):1285-93.

29. Kobel M, Weidensdorfer D, Reinke C, Lederer M, Schmitt WD, Zeng K, et al. Expression of the RNA-binding protein IMP1 correlates with poor prognosis in ovarian carcinoma. Oncogene. 2007;26(54):7584-9.

30. Walter O, Prasad M, Lu S, Quinlan RM, Edmiston KL, Khan A. IMP3 is a novel biomarker for triple negative invasive mammary carcinoma associated with a more aggressive phenotype. Hum Pathol. 2009;40(11):1528-33.

31. Fadare O, Liang SX, Crispens MA, Jones HW, 3rd, Khabele D, Gwin K, et al. Expression of the oncofetal protein IGF2BP3 in endometrial clear cell carcinoma: assessment of frequency and significance. Hum Pathol. 2013;44(8):1508-15.

32. Okada K, Fujiwara Y, Nakamura Y, Takiguchi S, Nakajima K, Miyata H, et al. Oncofetal protein, IMP-3, a potential marker for prediction of postoperative peritoneal dissemination in gastric adenocarcinoma. J Surg Oncol. 2012;105(8):780-5.

33. Kobel M, Xu H, Bourne PA, Spaulding BO, Shih Ie M, Mao TL, et al. IGF2BP3 (IMP3) expression is a marker of unfavorable prognosis in ovarian carcinoma of clear cell subtype. Mod Pathol. 2009;22(3):469-75.

34. Chen YL, Jeng YM, Hsu HC, Lai HS, Lee PH, Lai PL, et al. Expression of insulin-like growth factor II mRNA-binding protein 3 predicts early recurrence and poor prognosis in intrahepatic cholangiocarcinoma. Int J Surg. 2013;11(1):85-91.

35. Riener MO, Fritzsche FR, Clavien PA, Pestalozzi BC, Probst-Hensch N, Jochum W, et al. IMP3 expression in lesions of the biliary tract: a marker for high-grade dysplasia and an independent prognostic factor in bile duct carcinomas. Hum Pathol. 2009;40(10):1377-83.

36. Li D, Yan D, Tang H, Zhou C, Fan J, Li S, et al. IMP3 is a novel prognostic marker that correlates with colon cancer progression and pathogenesis. Ann Surg Oncol. 2009;16(12):3499-506.

37. Lin L, Zhang J, Wang Y, Ju W, Ma Y, Li L, et al. Insulin-like growth factor-II mRNA-binding protein 3 predicts a poor prognosis for colorectal adenocarcinoma. Oncol Lett. 2013;6(3):740-4.

38. Jiang Z, Chu PG, Woda BA, Rock KL, Liu Q, Hsieh CC, et al. Analysis of RNA-binding protein IMP3 to predict metastasis and prognosis of renal-cell carcinoma: a retrospective study. Lancet Oncol. 2006;7(7):556-64.

39. Jiang Z, Chu PG, Woda BA, Liu Q, Balaji KC, Rock KL, et al. Combination of quantitative IMP3 and tumor stage: a new system to predict metastasis for patients with localized renal cell carcinomas. Clin Cancer Res. 2008;14(17):5579-84.

40. Sitnikova L, Mendese G, Liu Q, Woda BA, Lu D, Dresser K, et al. IMP3 predicts aggressive superficial urothelial carcinoma of the bladder. Clin Cancer Res. 2008;14(6):1701-6.

41. Hu W, Yang Y, Xi S, Sai K, Su D, Zhang X, et al. Expression of CPEB4 in Human Glioma and Its Correlations With Prognosis. Medicine (Baltimore). 2015;94(27):e979.

42. Sun HT, Wen X, Han T, Liu ZH, Li SB, Wang JG, et al. Expression of CPEB4 in invasive ductal breast carcinoma and its prognostic significance. Onco Targets Ther. 2015;8:3499-506.

43. He X, Lin X, Cai M, Fan D, Chen X, Wang L, et al. High expression of cytoplasmic polyadenylation element-binding protein 4 correlates with poor prognosis of patients with colorectal cancer. Virchows Arch. 2017;470(1):37-45.

44. Zhou S, Wang GP, Liu C, Zhou M. Eukaryotic initiation factor 4E (eIF4E) and angiogenesis: prognostic markers for breast cancer. BMC Cancer. 2006;6:231.

45. Flowers A, Chu QD, Panu L, Meschonat C, Caldito G, Lowery-Nordberg M, et al. Eukaryotic initiation factor 4E overexpression in triple-negative breast cancer predicts a worse outcome. Surgery. 2009;146(2):220-6.

46. Modelska A, Turro E, Russell R, Beaton J, Sbarrato T, Spriggs K, et al. The malignant phenotype in breast cancer is driven by eIF4A1-mediated changes in the translational landscape. Cell Death Dis. 2015;6:e1603.

47. McClusky DR, Chu Q, Yu H, Debenedetti A, Johnson LW, Meschonat C, et al. A prospective trial on initiation factor 4E (eIF4E) overexpression and cancer recurrence in node-positive breast cancer. Ann Surg. 2005;242(4):584-90; discussion 90-2.

48. Yin X, Kim RH, Sun G, Miller JK, Li BD. Overexpression of eukaryotic initiation factor 4E is correlated with increased risk for systemic dissemination in node-positive breast cancer patients. J Am Coll Surg. 2014;218(4):663-71.

49. Li BD, Gruner JS, Abreo F, Johnson LW, Yu H, Nawas S, et al. Prospective study of eukaryotic initiation factor 4E protein elevation and breast cancer outcome. Ann Surg. 2002;235(5):732-8; discussion 8-9.

50. Diab-Assaf M, Abou-Khouzam R, Saadallah-Zeidan N, Habib K, Bitar N, Karam W, et al. Expression of eukaryotic initiation factor 4E and 4E binding protein 1 in colorectal carcinogenesis. Int J Clin Exp Pathol. 2015;8(1):404-13.

51. Furic L, Rong L, Larsson O, Koumakpayi IH, Yoshida K, Brueschke A, et al. eIF4E phosphorylation promotes tumorigenesis and is associated with prostate cancer progression. Proc Natl Acad Sci U S A. 2010;107(32):14134-9.

52. Chen CN, Hsieh FJ, Cheng YM, Lee PH, Chang KJ. Expression of eukaryotic initiation factor 4E in gastric adenocarcinoma and its association with clinical outcome. J Surg Oncol. 2004;86(1):22-7.

53. Nathan CO, Liu L, Li BD, Abreo FW, Nandy I, De Benedetti A. Detection of the proto-oncogene eIF4E in surgical margins may predict recurrence in head and neck cancer. Oncogene. 1997;15(5):579-84.

54. Inamdar KV, Romaguera JE, Drakos E, Knoblock RJ, Garcia M, Leventaki V, et al. Expression of eukaryotic initiation factor 4E predicts clinical outcome in patients with mantle cell lymphoma treated with hyper-CVAD and rituximab, alternating with rituximab, high-dose methotrexate, and cytarabine. Cancer. 2009;115(20):4727-36.

55. Zheng J, Li J, Xu L, Xie G, Wen Q, Luo J, et al. Phosphorylated Mnk1 and eIF4E are associated with lymph node metastasis and poor prognosis of nasopharyngeal carcinoma. PLoS One. 2014;9(2):e89220.

56. Fan S, Ramalingam SS, Kauh J, Xu Z, Khuri FR, Sun SY. Phosphorylated eukaryotic translation initiation factor 4 (eIF4E) is elevated in human cancer tissues. Cancer Biol Ther. 2009;8(15):1463-9.

57. Chao MW, Wang LT, Lai CY, Yang XM, Cheng YW, Lee KH, et al. eIF4E binding protein 1 expression is associated with clinical survival outcomes in colorectal cancer. Oncotarget. 2015;6(27):24092-104.

58. Rojo F, Najera L, Lirola J, Jimenez J, Guzman M, Sabadell MD, et al. 4E-binding protein 1, a cell signaling hallmark in breast cancer that correlates with pathologic grade and prognosis. Clin Cancer Res. 2007;13(1):81-9.

59. Qu Y, Zhao R, Wang H, Chang K, Yang X, Zhou X, et al. Phosphorylated 4EBP1 is associated with tumor progression and poor prognosis in Xp11.2 translocation renal cell carcinoma. Sci Rep. 2016;6:23594.

60. Castellvi J, Garcia A, Ruiz-Marcellan C, Hernandez-Losa J, Peg V, Salcedo M, et al. Cell signaling in endometrial carcinoma: phosphorylated 4E-binding protein-1 expression in endometrial cancer correlates with aggressive tumors and prognosis. Hum Pathol. 2009;40(10):1418-26.

61. Silvera D, Arju R, Darvishian F, Levine PH, Zolfaghari L, Goldberg J, et al. Essential role for eIF4GI overexpression in the pathogenesis of inflammatory breast cancer. Nat Cell Biol. 2009;11(7):903-8.

62. Tu L, Liu Z, He X, He Y, Yang H, Jiang Q, et al. Over-expression of eukaryotic translation initiation factor 4 gamma 1 correlates with tumor progression and poor prognosis in nasopharyngeal carcinoma. Mol Cancer. 2010;9:78.

63. Chen Y, Knosel T, Kristiansen G, Pietas A, Garber ME, Matsuhashi S, et al. Loss of PDCD4 expression in human lung cancer correlates with tumour progression and prognosis. J Pathol. 2003;200(5):640-6.

64. Gao F, Wang X, Zhu F, Wang Q, Zhang X, Guo C, et al. PDCD4 gene silencing in gliomas is associated with 5'CpG island methylation and unfavourable prognosis. J Cell Mol Med. 2009;13(10):4257-67.

65. Fassan M, Cagol M, Pennelli G, Rizzetto C, Giacomelli L, Battaglia G, et al. Programmed cell death 4 protein in esophageal cancer. Oncol Rep. 2010;24(1):135-9.

66. Mudduluru G, Medved F, Grobholz R, Jost C, Gruber A, Leupold JH, et al. Loss of programmed cell death 4 expression marks adenoma-carcinoma transition, correlates inversely with phosphorylated protein kinase B, and is an independent prognostic factor in resected colorectal cancer. Cancer. 2007;110(8):1697-707.

67. Wang X, Wei Z, Gao F, Zhang X, Zhou C, Zhu F, et al. Expression and prognostic significance of PDCD4 in human epithelial ovarian carcinoma. Anticancer Res. 2008;28(5B):2991-6.

68. Fassan M, Pizzi M, Battaglia G, Giacomelli L, Parente P, Bocus P, et al. Programmed cell death 4 (PDCD4) expression during multistep Barrett's carcinogenesis. J Clin Pathol. 2010;63(8):692-6.

69. Reis PP, Tomenson M, Cervigne NK, Machado J, Jurisica I, Pintilie M, et al. Programmed cell death 4 loss increases tumor cell invasion and is regulated by miR-21 in oral squamous cell carcinoma. Mol Cancer. 2010;9:238.

70. Wang S, Rosenwald IB, Hutzler MJ, Pihan GA, Savas L, Chen JJ, et al. Expression of the eukaryotic translation initiation factors 4E and 2alpha in non-Hodgkin's lymphomas. Am J Pathol. 1999;155(1):247-55.

71. Shi B, Hsu HL, Evens AM, Gordon LI, Gartenhaus RB. Expression of the candidate MCT-1 oncogene in B- and T-cell lymphoid malignancies. Blood. 2003;102(1):297-302.

72. Leprivier G, Remke M, Rotblat B, Dubuc A, Mateo AR, Kool M, et al. The eEF2 kinase confers resistance to nutrient deprivation by blocking translation elongation. Cell. 2013;153(5):1064-79.

73. Ruggero D, Grisendi S, Piazza F, Rego E, Mari F, Rao PH, et al. Dyskeratosis congenita and cancer in mice deficient in ribosomal RNA modification. Science. 2003;299(5604):259-62.

74. Marcel V, Ghayad SE, Belin S, Therizols G, Morel AP, Solano-Gonzalez E, et al. p53 acts as a safeguard of translational control by regulating fibrillarin and rRNA methylation in cancer. Cancer Cell. 2013;24(3):318-30.

75. Macari F, El-Houfi Y, Boldina G, Xu H, Khoury-Hanna S, Ollier J, et al. TRM6/61 connects PKCalpha with translational control through tRNAi(Met) stabilization: impact on tumorigenesis. Oncogene. 2016;35(14):1785-96.

76. Delaunay S, Rapino F, Tharun L, Zhou Z, Heukamp L, Termathe M, et al. Elp3 links tRNA modification to IRES-dependent translation of LEF1 to sustain metastasis in breast cancer. J Exp Med. 2016;213(11):2503-23.

77. Spinola M, Galvan A, Pignatiello C, Conti B, Pastorino U, Nicander B, et al. Identification and functional characterization of the candidate tumor suppressor gene TRIT1 in human lung cancer. Oncogene. 2005;24(35):5502-9.
